# SAFEGUARD: Transforming Military Suicide Prevention Through Predictive Analytics and Targeted Interventions

**DOI:** 10.1007/s11920-026-01664-6

**Published:** 2026-02-27

**Authors:** Thomas H. Nassif, Amy B. Adler, Robert D. Atkinson, Kate H. Bentley, Robin E. Bonomi, Jenny D’Olympia, Emily R. Edwards, Rebecca G. Fortgang, Brandon A. Gaudiano, Joseph C. Geraci, Marianne Goodman, Daniel Herman, Evan M. Kleiman, Sarah Maggio, Allison McDaniel, James A. Naifeh, Matthew K. Nock, Christopher Paine, Timothy Regan, John Torous, Vincent F. Capaldi, Ronald C. Kessler

**Affiliations:** 1 Department of Psychiatry, Uniformed Services University, Bethesda, MD USA; 2 Center for Military Psychiatry and Neuroscience, Walter Reed Army Institute of Research, Silver Spring, MD USA; 3 Department of Mental Health, Naval Medical Center Portsmouth, Portsmouth, VA USA; 4 Department of Psychiatry, Massachusetts General Hospital, Boston, MA USA; 5 Department of Psychiatry, Yale School of Medicine, New Haven, CT USA; 6 Counseling and Behavioral Health Department, William James College, Newton, MA USA; 7 Department of Psychiatry, Icahn School of Medicine at Mount Sinai, New York, NY USA; 8 VISN 2 Mental Illness Research, Education, and Clinical Center, Department of Veterans Affairs, Bronx, NY USA; 9 Department of Psychiatry & Human Behavior, Alpert Medical School of Brown University, Providence, RI USA; 10 Psychosocial Research Program, Butler Hospital, Providence, RI USA; 11 Hunter College and Graduate Center, City University of New York, New York City, NY USA; 12 Department of Psychology, Rutgers, The State University of New Jersey, Piscataway, NJ USA; 13 Henry M. Jackson Foundation for the Advancement of Military Medicine, Bethesda, MD USA; 14 Department of Psychology, Harvard University, Boston, MA USA; 15 Department of Medical and Clinical Psychology, Uniformed Services University, Bethesda, MD USA; 16 Division of Digital Psychiatry, Beth Israel Deaconess Medical Center, Harvard Medical School, Boston, MA USA; 17 Department of Health Care Policy, Harvard Medical School, Boston, MA USA

**Keywords:** Active-duty servicemembers, Suicide prevention, Predictive analytics, Targeted intervention, Precision medicine, Digital health technology

## Abstract

**Purpose of Review:**

Machine learning predictive modeling can support scalable prevention of suicide-related behavior (SRB). SAFEGUARD is a three-pronged universal, indicated, and clinical SRB-prevention intervention system focused on key military career touchpoints.

**Recent Findings:**

The targeted SAFEGUARD interventions are designed to improve on the mixed results of universal interventions. *Level Up* uses digital tools, personalized messaging, and remote booster sessions to deliver customized universal military-focused cognitive behavioral therapy skills training designed to reduce SRBs during first duty assignments. *Operation Life Force* delivers remote group dialectical behavior therapy skills training with a mental toughness focus to soldiers identified during annual physicals as high-risk for SRBs. *Pathfinding* delivers remote wrap-around case management after psychiatric inpatient discharge to soldiers identified as high-risk for SRBs.

**Summary:**

SAFEGUARD is a data-driven system for SRB prevention that delivers targeted best-practice interventions at critical points to optimize impact and efficiently use mental health resources across the military.

## Introduction

Although suicide rates among active-duty U.S. servicemembers were historically lower than those of similar civilian populations, this trend began to shift in the early 2000s. By 2008, suicide rates among military personnel began surpassing those in age- and gender-matched civilians, a trend that has continued into recent years [1–3]. In 2023, 523 servicemembers died by suicide, marking a 9% increase in suicides compared to the previous year [3]. This upward trend presents a significant challenge to the Department of War (DoW). Although the DoW has invested heavily in suicide prevention programs, the military continues to face difficulties in accurately identifying individuals at risk. Moreover, many effective strategies are resource-intensive, making it difficult to implement at scale across the entire force. Additionally, concerns about career repercussions often discourage servicemembers from seeking help. Therefore, there is a pressing need for innovative, evidence-based approaches to enhance suicide risk detection and provide timely, targeted interventions. One such initiative is SAFEGUARD (Suicide Avoidance Focused Enhanced Group Using Algorithm Risk Detection), a three-pronged universal, indicated, and clinical suicide-related behavior (SRB) prevention intervention system focused on key points in the military career that uses machine learning (ML) predictive modeling to target best-practice interventions to high-risk soldiers.

For over 50 years, suicide researchers have searched for a set of risk factors to reliably predict suicidal behavior. Despite these efforts, no consistent predictors have emerged [4]. Suicide is influenced by a complex interaction of psychological, social, biological, and environmental risk factors, each with relatively small effects [5]. Recognizing the limitations of traditional tools, the DoD’s Suicide Prevention Research Strategy (SPRS) for FY 2020–2030 called for leveraging big data and ML to develop predictive models based on existing administrative and health records [6].

A milestone in this effort was the Army Study to Assess Risk and Resilience in Servicemembers (Army STARRS), which used data from over 50 Army and DoW databases to develop ML models that were found to predict suicide-related behaviors (SRBs) at key phases in the military career [7]. This work laid the foundation for expanding understanding of risk-resilience factors for suicidality in the Army [8]. For example, the first year of military service, a critical time of transition when soldiers typically arrive at their first duty station, is known to be an especially high-risk period for SRBs among soldiers [9, 10]. The STARRS team showed that these early-career SRBs can be predicted with good accuracy using information collected in a STARRS New Soldier Survey (NSS) that was administered in Reception Battalion [11]. This proof-of-concept study demonstrated that routine administration of a NSS could be of value for targeted prevention planning.

One key challenge in suicide prevention is identifying at-risk individuals who have no formal mental health diagnosis or frequent healthcare interactions [6]. This problem is compounded by stigma and concerns about the professional consequences of seeking help, which can discourage many servicemembers from disclosing suicidal thoughts [12]. For example, Naifeh and colleagues (2025) found that 95% of suicide attempts occurred among soldiers who did not report suicidality during their mandatory Periodic Health Assessment (PHA) in the preceding six months [13]. But this study of PHA data also showed that incorporating DoW administrative data such as healthcare and service-related records into a ML model significantly improved prediction accuracy, making it possible to target high-risk preventive interventions in conjunction with PHAs across the entire military career [13].

STARRS has also successfully developed machine learning models that can identify soldiers at high risk for SRBs at other critical junctures of the military career, most notably during outpatient mental health treatment [14], after psychiatric inpatient discharge [15], and after transition out of active service [16, 17]. All these models use a wide range of administrative variables as predictors as well as employ a wide range of machine learning algorithms (e.g., penalized regression, adaptive splines, bagging and boosting tree algorithms, neural networks, support vector machines) combined in ensembles [18]. These models have shown that a significant concentration of risk can be found for SRBs. That is, a relatively small proportion of soldiers estimated by the models to be most likely to engage in future SRBs account for a high proportion of subsequently observed SRBs. For example, the STARRS model for SRBs after transitioning out of service found that the 10% of soldiers with highest predicted risk of SRBs accounted for nearly 45% of the SRBs that occurred in this population in the first year after separation [16].

While STARRS has been instrumental in identifying risk factors, applying these insights to widespread suicide prevention across the DoW remains a challenge. Effective suicide prevention requires timely, coordinated interventions that address a servicemember’s psychological, environmental, and structural risk factors during high-risk periods. The right intervention depends on the period in the military career when the intervention is being delivered as well as on the characteristics of the soldiers selected to participate in the intervention. The initial SAFEGUARD intervention suite is designed to span the range of risk during the Army career and to implement previously developed best-practice interventions that are appropriately modified for the special needs of Army soldiers at the points in the military career selected based on the epidemiological results from STARRS.

The three initial SAFEGUARD interventions are described in the current article. As detailed below, they include: (1) a universal intervention with features that can be customized to the need of each participant delivered in a group orientation session on arrival at a first duty assignment and continuing with lessons delivered through guided app-based skills training over the next months and remote online group booster sessions delivered over the remainder of the first year of service; (2) an indicated remote online five-session group intervention designed to be delivered to soldiers identified by a ML model at the time of annual PHAs to be high-risk for SRBs; and (3) a one-on-one case manager intervention designed to be delivered over a period of six months after psychiatric inpatient discharge to soldiers identified by ML to be high-risk for SRBs.

It is important to note that these three interventions and the three points in the military career in which they are being delivered are not the only ones of potential importance for preventing SRBs in this population. Rather, they were selected to illustrate the way in which the unique collection of administrative systems available in the DoW can be used to target high-risk soldiers at key touchpoints and evidence-based interventions appropriate for those touchpoints can be selected, modified for military use, and implemented to reduce SRBs across the military career. Each of the three interventions has been modified and implementation science considerations have been addressed prior to launching a pragmatic experimental evaluation of the intervention in fiscal years 2026–2028 in which reduction in SRBs is the primary outcome. It is noteworthy that although we did not implement interventions for high-risk servicemembers after separation from service even though STARRS ML models exist that target transitioning servicemember at high risk of subsequent SRBs [19], a program using these models is underway in the Veterans Health Administration (VHA) [20].

**Fig. 1:**
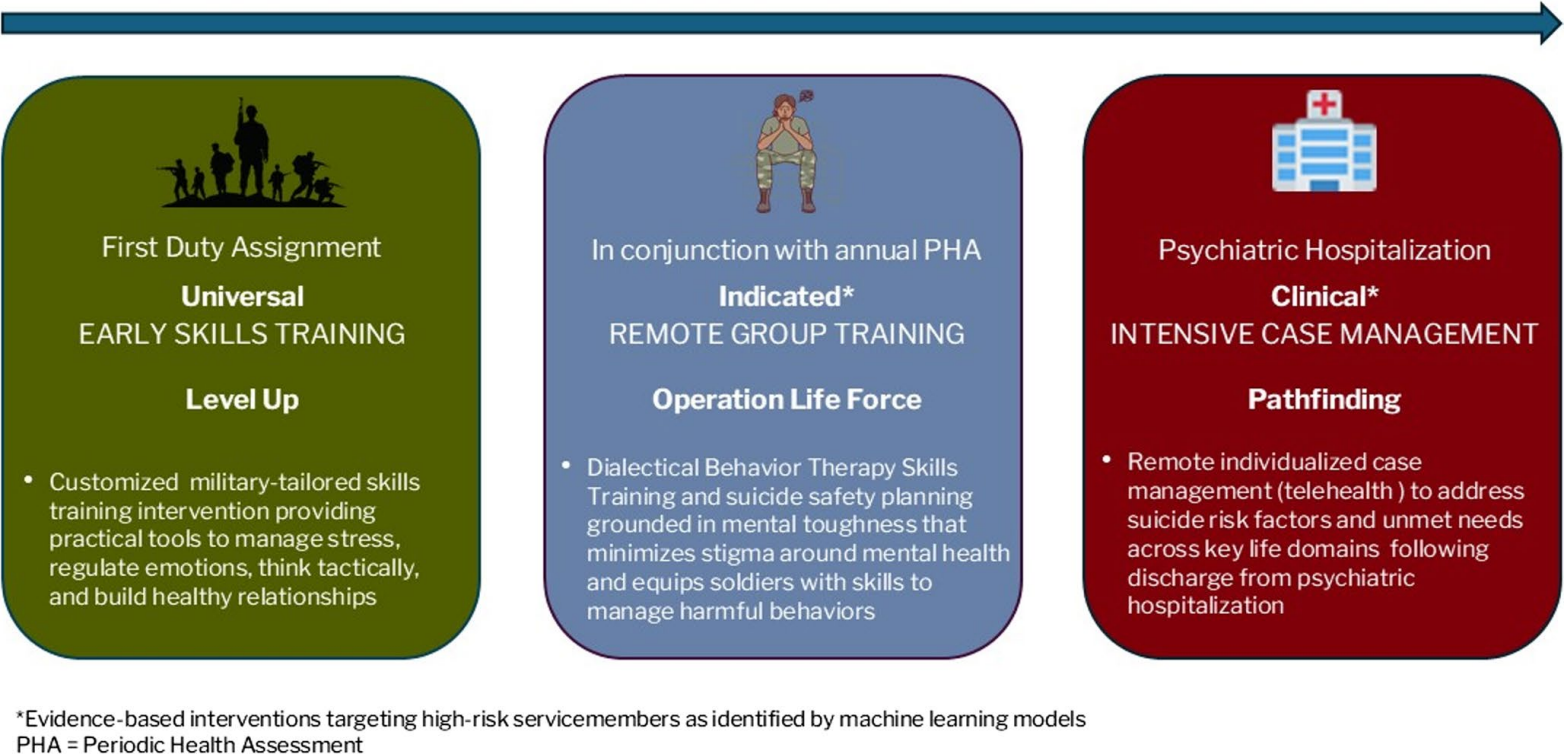
SAFEGUARD's three-pronged approach to target suicide prevention at universal, indicated , and clinical levels. *Evidence-based interventions targeting high-risk servicemembers as identified by machine learning models PHA=Periodic Health Assessment

SAFEGUARD’s three-pronged approach targets suicide prevention at different levels: universal, indicated, and clinical (Fig. 1). The universal intervention, *Level Up*, targets new servicemembers arriving at their first duty station—a period characterized by isolation and adjustment stress. The indicated intervention, *Operation Life Force*, uses predictive analytics to identify at-risk servicemembers throughout their careers and delivers group-based skills and prevention strategies. Lastly, the clinical intervention, *Pathfinding*, focuses on high-risk individuals following psychiatric inpatient discharge and provides individualized case management to high-risk individuals following psychiatric hospitalization, a period of heightened suicide risk (Fig. 1).

By aligning precision risk identification with tailored interventions at critical touchpoints, SAFEGUARD aims to optimize mental health resource allocation and enhance coordination between medical and non-medical systems. Ultimately, the goal is to develop a sustainable, scalable framework for suicide prevention that can be implemented across the active-duty force. Here, we provide an overview of these three SAFEGUARD interventions. 

## Universal Prevention among Early Career Servicemembers

### Prior Universal Prevention Efforts

Universal prevention programs aim to reduce the risk of mental health problems and related adverse outcomes—such as suicide, substance abuse, and interpersonal violence—before they emerge. One widely used approach is life skills training, which focuses on building psychosocial competencies like problem-solving, decision-making, emotion regulation, and drug resistance strategies to help individuals navigate challenging transitions and reduce vulnerability to harmful behaviors.

Originally developed for high school students transitioning to adulthood, early life skills programs were shown to increase positive outcomes (e.g., educational and occupational success) and reduce negative ones (e.g., mental illness, substance abuse, violence) [21–24]. More recently, universal programs have incorporated cognitive behavioral therapy (CBT), mindfulness, social-emotional learning, and related approaches to prevent mental health disorders and improve well-being in adolescents and young adults [25, 26]. However, findings have been mixed. Some studies show meaningful preventive effects [27], whereas others find no impact [28, 29], and a few find unintended (iatrogenic) effects [30, 31].

These inconsistencies may be due to methodological issues, such as small sample sizes, limited experimental rigor, or barriers to active participation in the intervention. Many programs are implemented in classroom settings, which may discourage honest engagement and participant application of the skills to real-world stressors. Additionally, skills use often declines after structured training ends and most programs did not offer booster opportunities for continued engagement. Finally, the skill level of trainers may have limited program effectiveness. Recent studies suggest long-term engagement and effectiveness can be enhanced by incorporating interactive digital tools [32], personalized messaging [33], coaching support [34] and ongoing booster sessions [35].

## The Level Up Intervention

To address the need for effective universal prevention for new servicemembers, our team developed *Level Up: Boost Your Life Skills (“Level Up”)*: a strengths-based, military-tailored intervention. Designed specifically for soldiers arriving at their first duty assignment, *Level Up* aims to equip them with practical tools to manage stress, regulate emotions, think tactically, and build healthy relationships—skills critical to performance, readiness, and well-being.

*Level Up* was created by a user-centered design process, incorporating feedback from servicemembers, behavioral health experts, and digital intervention developers, along with strong support from military leadership at the test site including the commander of the incoming reception command and the commanding general of the military installation. The program integrates evidence-based psychosocial strategies with delivery methods optimized for engagement and real-world use during a soldier’s first year on duty. Grounded in principles from CBT [36], acceptance and commitment therapy (ACT) [37], problem-solving therapy [38], motivational enhancement [39], and performance psychology [40], the content is tailored to military culture using relevant examples, relatable language, and engaging skill names. The goals of the *Level Up* program are to equip new soldiers with evidence-based skills in domains key to readiness, performance, and well-being, including stress management, emotion regulation, tactical thinking, and healthy relationships (Table 1).

**Table 1:** *Level Up*: overview of key skills content

Domain	Rationale	Example Skills
Stress Management	Effectively responding to stress and other negative emotions maximizes readiness and enhances performance	1. Compass of Control 2. Boost Your Focus/Lock In 3. Break Through Anger
Tactical Thinking	Strategically responding to thoughts and feelings influences performance and readiness	1. Flip Your Mindset 2. Thought Labeling and Distancing 3. Give Yourself Grace/Ditch the Doubt
Goals and Plans	Goal-setting, problem-solving, and task management can support personal and professional development	1. Tactical Problem-Solving 2. Turn Ideas into Action/Set SMART Goals 3. Become a Leader
Healthy Routines	Maintaining physical and mental health can optimize performance and overall well-being, especially during transitions, and adapting healthy routines to a variety of settings (e.g., in the field, deployment, combat, congregate housing)	1. Sleep, Nutrition, Substance Use 2. How to Build a Habit 3. What Went Right?/Gratitude Practice
Healthy Relationships	Fostering meaningful and healthy interpersonal connections are key to developing personal and professional attributes	1. Build Healthy Connections 2. Boost Communication, Navigate Conflict 3. Battle Buddy Boost/Be a Good Battle
Crisis and Other Resources	Making resources readily accessible when needed can reduce the risk of adverse outcomes	1. Crisis Survival Skills and 24/7 Resources 2. Managing Money: Tips and Resources 3. Parenting: Tips and Resources

*Level Up* includes four core components:

1. Introductory Group Session (90 min): Delivered live, this session motivates engagement in the program and introduces soldiers to key skills. It also provides guidance on installing and using the *Level Up* mobile app.
2. Interactive Mobile App: Available for up to one year, the app features brief, engaging self-guided lessons and practice activities using text and video. Daily prompts, reminders, progress tracking, and leaderboards help reinforce engagement.
3. Personalized Digital Messaging: For the first four weeks, soldiers receive interactive support and encouragement from a trained skills coach through secure in-app messaging.
4. Booster Sessions: Virtual, drop-in group sessions are offered throughout the year, each focusing on a key life domain (e.g., financial literacy, relationships, leadership, stress).

By combining live group sessions, digital tools, personalized messaging and booster sessions, *Level Up* is designed to increase engagement, promote long-term skill use, and reduce the risk of suicide and other adverse outcomes among new servicemembers. The *Level Up* content overlaps somewhat with the older Comprehensive Soldier and Family Fitness (CSF2) program, but features a fundamentally different delivery mechanism that uses modern digital delivery mechanisms to connect directly with the soldier and overcome the barriers to delivery that existed in CSF2 [41, 42].

## Targeted Suicide Prevention among At-Risk Servicemembers

### Prior Suicide Prevention Efforts

Two interventions with the strongest evidence for decreasing suicide attempts include dialectical behavior therapy (DBT) [43] and suicide safety planning [44]. DBT helps individuals develop and strengthen coping skills, improve emotion regulation, and increase social connections, especially during a crisis. Although demonstrated to be effective with servicemembers and veterans [45–47], DBT is time-intensive and costly to administer, limiting scalability. In contrast, suicide safety planning is a brief, low-cost intervention, typically delivered in a single 30-minute session. Through suicide safety planning, individuals identify sources of internal (e.g., coping strategies) and external (e.g., support persons) support to use in a moment of crisis [44]. While proven to be effective, safe, and widely adopted [48], its effectiveness depends on the personal relevance of the supports identified by the at-risk individual [49].

Building on the strengths of DBT [43] and suicide safety planning [44], our team developed and tested Project Life Force (PLF), a group-based intervention that was designed to help high-risk patients in the VHA to develop effective skills and strategies for managing crisis situations and maps these techniques onto steps of a suicide safety plan [45, 50]. Research indicates that PLF is effective in decreasing suicide risk in veteran populations [45, 50].

However, PLF’s explicit focus on suicidal thoughts and behaviors as applied to veteran populations presents challenges to implementation in active-duty military contexts, where stigma around suicide disclosure remains high. To address these barriers and improve scalability within the military, PLF was restructured into a culturally relevant, training-based program—*Operation Life Force* (*OLF*). In doing so, we worked closely with the director of psychological Health at the test site and obtained strong support from military leadership including the commanding general of the military installation.

### The Operation Life Force Intervention

*OLF* retains the therapeutic foundation of DBT and suicide safety planning but reframes the content through the lens of mental toughness—a performance-oriented concept derived from elite athletic performance that emphasizes consistently high-level performance in challenging situations [51]. By emphasizing resilience, focus, strength, and mental toughness in a format that is culturally and operationally relevant to soldiers, and congruent with valued masculine norms (e.g., strength, responsibility, being a good partner), *OLF* aims to resonate more strongly with military culture and reduces stigma associated with mental health or suicide-related interventions. Linking mental health care with valued masculine norms in this way has been shown in previous research to increase the engagement of young men with efforts to provide psychological help [52].

Mental toughness is defined as the ability to effectively use individual skills and talents to their fullest potential, regardless of the circumstances. Mental toughness is best understood using the three dimensions of hardiness, initially conceived as commitment, control, and challenge [53], and later adapted to include confidence in the 4 C’s model [54]:

1. Challenge — the extent to which one views problems as opportunities for self-development.
2. Commitment — consistency and sustained focus in activities.
3. Control — emotional regulation under stress.
4. Confidence — belief in oneself and others.

Previous research shows that mental toughness is strongly associated with protective factors for suicide, including psychological wellbeing [55], quality of life [56], and perceptions of stress [57]. In addition, studies on mental toughness in sports [54], business [58], police [56, 57], and military populations [59, 60] demonstrate that mental toughness can be enhanced through intervention [61]. Our team added a fifth “C” to this model, Connection, to emphasize the importance of social support.

*OLF* follows a similar format as DBT and the PLF manualized group intervention for high-risk suicidal veterans [45, 50]. This format encourages group cohesion, supports practice of emotion regulation skills/strategies, fosters learning between soldiers, mitigates social isolation, and helps to reshape maladaptive beliefs about failure, perceived weaknesses/strengths, anger, and help-seeking. However, instead of developing a suicide prevention plan, *OLF* participants build a mental toughness action plan. The emphasis on mental toughness and de-emphasis on suicide also encourages soldiers to apply skills learned through *OLF* to manage a wide range of harmful behaviors that may interfere with military service performance. Further, by explicitly grounding the “mental toughness” language within the therapeutic architecture of PLF, *OLF* offers a narrative that resonates with soldiers’ perceived needs for strength and mental endurance while simultaneously providing a skills-based training that aims to impact suicidal ideation and behaviors.

*OLF* is delivered in a seven-module group training format, offered both with in-person and virtual settings, during which a personalized Mental Toughness Action Plan is progressively constructed to serve as a “playbook” for recalling specific training skills (Table 2). The program targets soldiers who are identified at the time of their Periodic Health Assessment as being at comparatively high risk of an SRB based on an expansion of the ensemble machine learning developed for this purpose in STARRS using predictors drawn from Army and DoW administrative data systems [13]. The model is integrated into the Military Health System Information Platform, allowing daily model implementation so that there is no delay in *OLF* Recruitment. The program systematically advances through various dimensions of mental toughness, with each module building upon preceding ones. The acronym, GRIND, is used to introduce and organize each of the 5 C’s components. Foundational principles of DBT underlie each GRIND component, particularly in its prophylactic role against suicide. The GRIND model includes: *G*row Through Support (Module 7), *R*elentlessness (Module 4), *I*nvite Adversity (Module 5), *N*onstop Resolve (Module 6), *D*ominate your emotions (Module 3).

**Table 2:** *Operation life force* (*OLF*) session: overview of key skills content

Module Number	Session Focus	OLF Skill Covered	Associated DBT Skill
1	Introduction, define Mental Toughness, introduce GRIND framework	GRIND Framework	
2	Identification of obstacles to Mental Toughness	Recognizing Emotions, Triggers, Urges, Addressing Your Thoughts	Recognition of Warning Signs
3	**Dominate Your Emotions** - controlling emotions, breaking down intervention points and how to respond instead of react	Emotion regulation, responding with control, mastering mental reset tools	DBT Emotion Regulation skills
4	**Relentlessness** - mastering the ability to push forward past the point of quitting	Discipline, skills to help face discomfort	DBT Distress Tolerance skills
5	**Invite Adversity** - fail forward, seeing failure as an opportunity for growth	Reframing failures, skills that emphasize learning, adjusting, and improving	DBT Cognitive Restructuring skills
6	**Nonstop Resolve** - owning the setback, taking accountability of mistakes to reset and move forward	Learning skills to accept mistakes and move forward	DBT Acceptance skills
7	**Grow Through Support** - building the inner circle and recognizing when to lean on that support	Interpersonal effectiveness and skills asking for help	DBT Interpersonal Effectiveness skills

GRIND stands for “Grow Through Support,” “Relentlessness,” “Invite Adversity,” “Nonstop Resolve,” and “Dominate your emotions

OLF = Operation Life Forc

DBT = Dialectical behavior therapy

Each module combines didactic content, video examples, group discussion, and skills practice. Soldiers are encouraged to apply skills to real-life situations, share personal experiences, and reflect on how mental toughness applies to their military roles. Training materials are available in both digital (web-based) and hardcopy formats to accommodate diverse learning environments. *OLF* adapts proven suicide prevention strategies into a military relevant, strengths-based training focusing on mental toughness to equip soldiers with tools that support mental health and mission readiness.

### Early Intervention Following Psychiatric Inpatient Discharge

#### Prior Suicide Prevention Efforts

Psychological autopsy studies, including those conducted within the U.S. military, show that most individuals who die by suicide have a psychiatric disorder [62, 63]. Among those receiving treatment, the highest risk period is immediately after psychiatric inpatient discharge [64–66]. Though only about 1% of U.S. adults are discharged from psychiatric hospitalization each year, they account for approximately 14% of all suicides [66]. In the U.S. Army, the suicide risk within the next 12 months after psychiatric inpatient discharge is over 14 times as high as in the broader Army population [15].

Early intervention beginning either before or shortly after psychiatric inpatient discharge can reduce suicide-related outcomes [67–69]. For example, brief supportive contact interventions and brief CBT for suicide prevention have been shown to reduce suicidal ideation, suicide attempts, and emergency department visits [70, 71]. However, universal application of these approaches would likely exceed the capacity of most health systems. As a result, efforts have turned toward predictive analytics to identify high-risk individuals who might benefit most.

STARRS researchers developed a machine learning model to predict suicide risk after psychiatric inpatient discharge based on predictors extracted from administrative systems and using an ensemble estimation approach that was later replicated in the Department of Veterans Affairs [15, 72]. The extensive administrative variable prediction battery, detailed in the published reports, is currently being used to guide a randomized trial in the Veterans Health Administration where veterans at highest predicted risk are assigned to receive either treatment as usual (TAU) or TAU plus an intensive case management intervention known as CLASP (Coping with Long Term Active Suicide Program) [73]. CLASP has been shown to have significant aggregate effects on SRBs including fewer suicide attempts within one year following psychiatric hospital discharge [74]. However, further research is needed to apply this model and intervention to active-duty populations. This was accomplished by updating the ensemble model for the Army to specifically target participants in the SAFEGUARD Pathfinding trial for the Army to do this in targeting participants in the SAFEGUARD *Pathfinding* trial. This updated model was integrated into the Military Health System Information Platform, allowing daily model implementation as soldiers experience psychiatric inpatient discharge so that there is no delay in *Pathfinding* recruitment.

## Pathfinding Intervention

Building on the success of CLASP, we developed *Pathfinding*, a six-month, manualized intervention that integrates elements from two evidence-based case management models—CLASP and Critical Time Intervention (CTI)—and adapts them for the unique needs of a military population. CLASP is an adjunctive case management intervention for suicidal patients during transitions in care, including the transition from psychiatric hospitals back to the community [74]. CLASP supports suicidal individuals during high-risk transitions using two core therapeutic strategies:

1. Acceptance and Commitment Therapy (ACT), which helps individuals clarify personal values and set goals aligned with those values [75]
2. Family Intervention: Telephone Tracking (FITT), which involves regular phone contact with patients and their support persons to provide support in key areas relevant to the patient's health [76, 77].

CTI, originally developed for individuals leaving restrictive settings (e.g., foster care, prisons, psychiatric hospitals), focuses on strengthening connections to long-term community support to facilitate reintegration, promote continuity of care, and prevent relapse [78]. *Pathfinding* also incorporates elements from HEARTH (Help with Employment, Agency, Risk, Transitions and Housing), a CTI-based intervention that addresses post-separation risks such as homelessness, unemployment, and suicide among veterans [79]. In addition to civilian populations, these types of transitions are particularly important in military populations as many servicemembers who experience psychiatric inpatient discharge are medically separated or administratively released from duty within a year.

In *Pathfinding*, each participant is paired with a master’s-level, licensed mental health professional, referred to as a “Guide,” who delivers structured, individualized support. These Guides are distinct from the participant’s clinical care team and focus on key suicide risk factors such as treatment engagement, social connection, problem-solving, and addressing unmet needs across key life domains (e.g., legal, financial, housing, and relationships; Table 3).

**Table 3:** *Pathfinding*: overview of key skills content

Domain	Rationale	Components
Orientation & Assessment	Effective engagement necessitates clarifying roles and comprehensively assessing the soldier’s risks and needs	1. Orientation to *Pathfinding* and Guide role 2. Collaborative Safety Plan development
Risk and Needs Assessment: Areas of Need and Values Alignment	Addressing underlying needs and values is crucial for effectively integrating risk factors and protective factors, thereby fostering a robust safety net and promoting sustained positive outcomes	1. Needs addressed include housing, financial, legal, relationships, readjustment, behavioral health, parenting/caretaking, work, spirituality, physical health 2. Values include family, relationships, friends, work, education, recreation, spirituality, physical/self care
Mission Planning & Goal Setting	Mission Plan development builds on identification of needs, and connection to the soldier’s unique values, empowering the soldier with concrete action steps to ensure progress	1. Development of the “Mission Plan” 2. Collaborative articulation of action steps 3. Enlisting support towards goal completion
Guided Case Management	Structured, time-limited support for practical needs during transitions reduces stressors and enhances stability, aligning with CTI & HEARTH principles	1. Ongoing case management for identified needs 2. Linkage to resources such as employment, housing, healthcare, community service
Crisis & Safety Protocols	Proactive safety measures, including robust safety planning and immediate support access, mitigating acute risk and ensuring well-being aligning with CLASP principles	1. Activation of suicide prevention hotlines 2. Safer Storage practices 3. Continuous risk assessment and triage
Integrated Support Person (SP)	Reducing isolation and boosting goal adherence, a support person (e.g., family or friend) attends sessions, aids in safety planning, and provides encouragement, with education for effective, non-intrusive support	1. Engaged through the intervention with joint contacts 2. Provide support for Mission Plan adherence 3. Provided with psychoeducation for support
Sustained Engagement & Follow-up	Post-intervention connection and progress assessment ensure continued support, identify risks, and facilitate long-term positive outcomes, reflecting CLASP and CTI principles	1. Final assessment of goal progress at 6 months 2. Variable SP participation and analysis 3. Follow-up surveys at 6 and 12 months after psychiatric inpatient discharge

*Pathfinding* consists of three phases: Mobilizing, Sustaining, and Maintaining. The Mobilizing phase begins as soon as possible after psychiatric inpatient discharge and includes weekly telehealth sessions during the first month. The initial focus of this phase is to orient participants to *Pathfinding*, develop goals for life after psychiatric inpatient discharge, and identify a support person (e.g., family member or friend). Guides work with participants to create a Mission Plan that identifies the participant’s values, goals, corresponding action steps, and relevant resources/referrals. This process is aided by the Servicemember Support Prioritization Tool, a structured worksheet that helps identify and prioritize areas of concern.

During the Sustaining and Maintaining phase, participants subsequently receive up to ten brief telehealth sessions (15–30 min each), with frequency of contacts diminishing over time as participants build autonomy and self-sufficiency. These sessions continue to focus on collaborative safety planning, needs and values assessment, mission planning and goal setting, guided case management, and sustained engagement with behavioral health treatment.

*Pathfinding* also includes support persons, which may consist of family members, friends, or mentors identified by the participant to support them through the intervention. The integration of support persons into each *Pathfinding* phase is especially important given that patients are often socially isolated after psychiatric inpatient discharge, sometimes in conjunction with being shunned by other servicemembers in reaction to the hospitalization. Support persons are contacted for up to five sessions (10–15 min each) to help monitor the participant’s clinical status, risk level, and Mission Plan progress, and to provide support and guidance to the support person in helping them be of optimal value to the patient.

Guides coordinate with participants’ treatment providers and document sessions in the electronic health record to enhance coordination of care. The result is a robust intervention framework designed to complement routine outpatient care following psychiatric inpatient discharge to decrease suicide risk and improve long-term outcomes among active-duty servicemembers.

### Coordination among and Expansion of the SAFEGUARD Interventions

As noted in the introduction, SAFEGUARD builds upon and improves earlier efforts at predictive suicide prevention by focusing on high-risk periods across a servicemember’s military career with tailored, evidence-based interventions targeting high-risk servicemembers as identified by ML models. The SAFEGUARD models are most directly comparable in this regard to the Veterans Affairs’ (VA) REACH VET program, a flagship initiative of VA based on continuously updating ML models to identify veterans at the highest risk for suicide to direct support from all veterans who were in contact with the VHA in the past 24 months [80]. The SAFEGUARD models, in comparison, are disaggregated. This change was based on evaluations showing that REACH VET has not led to a significant reduction in veteran suicide deaths [81, 82]. One potential reason for this failure is the broad scope and diverse interventions administered, which may hinder opportunities for providing targeted, effective interventions.

SAFEGUARD was designed to address these limitations through a fundamentally different approach from REACH VET that develops separate predictive models for different touchpoints in the military career and uses tailored interventions designed for purpose to address the needs of soldiers at these periods. This disaggregated approach offers two advantages over REACH VET. First, disaggregation leads to increased model performance accuracy by allowing more fine-grained administrative predictors to be used than in the aggregate REACH VET model. For example, the VA version of the model used in *Pathfinding* [72] identified nearly three times as many at-risk individuals who went on to die by suicide as the REACH VET model although the same number of people were targeted in both models [14, 72].

Second, unlike REACH VET, which has no special program it implements but merely identifies veterans estimated to be high-risk and relies on VHA Suicide Prevention Coordinators (SPC) to reach out and implement any intervention the SPC considers appropriate, SAFEGUARD pairs each high-risk touchpoint with a specific evidence-based intervention tailored to that touchpoint. We then provide targeted training and supervision to the individuals delivering that intervention to ensure intervention fidelity. This targeting is only aspirational in *Level Up*, as the initial implementation of this intervention is universal. But we plan to use ML trial emulation methods [83] in conjunction with the baseline survey data we obtain from each *Level Up* participant to analyze the results of the initial *Level Up* experiment to create some customization of intervention components for different soldiers in subsequent replications.

All SAFEGUARD interventions are grounded in strong theoretical and empirical foundations and powered by the rich administrative and electronic health record data systems available in the DoW. In *OLF* and *Pathfinding*, predictive analytics models based on these data sources are used to select participants. *Level Up*, although initially universal in design, will also evolve using post hoc analytics to selectively target new soldiers for more intensive outreach and personalized content in follow-on efforts. This precision treatment strategy will leverage both administrative data and self-report surveys to align intervention components with individual needs [83]. A key innovation across SAFEGUARD interventions is the use of a digital delivery platform, designed to maximize scalability, fidelity, and cost-effectiveness. All three interventions are delivered through a common mobile telehealth platform to maximize reach across a variety of military settings [84].

The most advanced digital integration is currently in *Level Up*, which uses the mindLAMP app [85] to deliver psychosocial skills training designed to enhance soldier mental health and readiness. Developed by a SAFEGUARD collaborator, mindLAMP is a highly customizable and flexible digital mental health platform that enables advanced technical and scientific features that have led it to be widely adapted by an international consortium of users [86]. Key mindLAMP features, such as synthesis of branching logic, modular organization of activities, activity favoriting and metric tracking, and in-app messaging and communication integrations with human support, all facilitate personalization and engagement of content delivery. These flexible mindLAMP delivery components can be refined over time to support *Level Up* and other subsequent SAFEGUARD interventions.

To ensure rigorous evaluation and enable comparison of results, SAFEGUARD uses parallel research designs, measurement instruments, and data analysis frameworks. All three studies employ similar baseline self-report assessments that include information about a wide range of evidence-based effect modifiers as well as information about history of SRBs. All three use archival baseline measures from the same integrated Army administrative data systems to ensure equivalence between intervention and control groups. Both self-report and administrative records of SRBs in the 12 months after randomization will be used as primary outcomes. All evaluations will employ best-practice analytic methods to maintain consistency and comparability in assessing effectiveness across interventions. This will feature use of a doubly robust machine learning method to estimate aggregate intervention effects by balancing differences in informative baseline covariates and adjusting for loss to follow-up using both a propensity score model (which predicts loss to follow-up within each arm as a function of baseline variables) and an outcome model (which predicts the outcome based on covariates available at baseline within the treatment arm given observation of the outcome) [87]. In addition, we will use an expansion of this doubly robust method to estimate heterogeneity of intervention effects [88]. Although the current focus of SAFEGUARD is on Army populations, if found to be effective, the goal will be to adapt these interventions and models to other military branches including the U.S. Navy, Air Force, and Marines.

It is important to acknowledge that while the ML targeting models used in *OLF* and *Pathfinding* are designed to identify soldiers with the greatest need for intervention, they do not guarantee that these soldiers are the ones most likely to benefit from the interventions. Nor do the models ensure that the selected interventions are the most effective options for each targeted individual, even though all interventions are evidence-based. The SAFEGUARD trials will assess whether these interventions are as effective for the high-risk Army soldiers currently receiving them as they were in earlier samples where they showed positive results. If the interventions prove less effective in these new populations, it will be important to explore and compare alternative approaches. Additionally, all three SAFEGUARD trials will include precision treatment analyses to examine whether certain soldiers respond more favorably than others, helping us better match interventions to individual needs in the future [89].

This focus on precision treatment will address the issue of what has come to be referred to as “fairness” in artificial intelligence, whereby ML models can support equitable delivery of care so that the right interventions are given to the right people [90]. This kind of fairness cannot be guaranteed in the first iteration of an intervention system. However, post hoc analysis can be carried out to determine which segment of the high-risk population is helped and which segment is not helped by the intervention deployed. Subsequently, follow-up efforts can be used to investigate alternative interventions for the latter segment of the high-risk population. Or, in the case of *Level Up*, with its many components, post hoc analysis can be carried out to investigate which components are of most value to which soldiers, so that approaches can be developed in subsequent iterations to ensure these different components are delivered optimally in a more customized approach than in the initial implementation.

## Conclusions

By establishing a sustainable and scalable framework that integrates predictive analytics, digital tools, and iterative refinement of intervention strategies, the SAFEGUARD initiative seeks to reduce the burden on military healthcare providers while expanding its reach to servicemembers at identified points of high-risk. This approach holds promise not only for initial implementation but also for future expansion to other high-risk periods in a servicemember’s career, such as deployment, relocation, and separation from service. Through precision risk identification and the delivery of tailored interventions across universal, indicated, and clinical levels, SAFEGUARD offers a transformative model for optimizing mental health resource allocation and improving coordination between medical and non-medical systems of care. The lessons learned from these three coordinated interventions may help to inform interventions with other high-risk occupations like policing and firefighting and be adapted for wider implementation in civilian communities as well, particularly targeting points of transition and other high-risk opportunities for intervention. 

## Key References

- Bilden R, Torous J. Global Collaboration Around Digital Mental Health: The LAMP Consortium. J Technol Behav Sci. 2022;7(2):227-33.
  - **○ ** This paper describes how an international digital intervention network based on the mindLAMP digital intervention platform, the platform used in SAFEGUARD, came into being and developed. This network represents a useful model for the potential expansion of the SAFEGUARD network as the three initial interventions are refined and new interventions are developed and implemented.
- Botvin GJ, Kantor LW. Preventing alcohol and tobacco use through life skills training. Alcohol Res Health. 2000;24(4):250-7.
  - **○ ** This paper details the development of a foundational Life Skills Training program, a school-based approach aimed to prevent substance use among youth by teaching key self-management and social skills.
- Calvert E, Cipriani M, Chen K, Dhima A, Burns J, Torous J. Evaluating clinical outcomes for anxiety and depression: A real-world comparison of the digital clinic and primary care. J Affect Disord. 2025;377:275-83.
  - **○ ** This paper shows how apps, specifically the one used in this effort, can translate precision and risk prediction research into tools used in routine care.
- Goodman M, Sullivan SR, Spears AP, Dixon L, Sokol Y, Kapil-Pair KN, et al. An Open Trial of a Suicide Safety Planning Group Treatment: “Project Life Force”. Archives of Suicide Research. 2021;25(3):690-703.
  - **○ **Operation Life Force, our indicated intervention for at-risk Army personnel, is an adaptation of Project Life Force, a virtual manualized safety-planning plus DBT skills group for high-risk suicidal veterans.
- Kessler RC, Luedtke A. Pragmatic Precision Psychiatry-A New Direction for Optimizing Treatment Selection. JAMA Psychiatry. 2021;78(12):1384-90.
  - **○ ** This paper presents a framework for using a rich network of administrative data such as that available in the Army to develop machine learning models both to target interventions and to investigate intervention effects. This framework is being used to guide all the advanced analytics employed in the SAFEGUARD initiative.
- Kessler RC, Warner CH, Ivany C, Petukhova MV, Rose S, Bromet EJ, et al. Predicting suicides after psychiatric hospitalization in US Army soldiers: the Army Study To Assess Risk and rEsilience in Servicemembers (Army STARRS). JAMA Psychiatry. 2015;72(1):49-57.
  - **○ ** This study used administrative data and machine-learning techniques to predict suicide risk among U.S. Army soldiers in the 12 months following psychiatric inpatient discharge, finding that the majority of suicides occurred among a small subset of hospitalizations with the highest predicted risk.
- Lin Y, Mutz J, Clough PJ, Papageorgious KA. Mental Toughness and Individual Differences in Learning, Educational and Work Performance, Psychological Well-being, and Personality: A Systematic Review. Frontiers in Psychiatry. 2017;8.
  - **○ **This systematic review of empirical studies describes associations between the construct of mental toughness and individual differences in learning, educational and work performance, psychological well-being, personality, and other psychological attributes, laying the groundwork for interventions that enhance mental toughness.
- Naifeh JA, Edwards ER, Bentley KH, Gildea SM, Kennedy CJ, King AJ, et al. Predicting suicide attempts among US Army soldiers using information available at the time of periodic health assessments. Nat Ment Health. 2025;3(2):242-52.
  - **○ **This article developed models to predict 6-month nonfatal and fatal suicide attempts using self-reported suicidality and Army administrative data.
- Torous J, Bucci S, Bell IH, Kessing LV, Faurholt-Jepsen M, Whelan P, et al. The growing field of digital psychiatry: current evidence and the future of apps, social media, chatbots, and virtual reality. World Psychiatry. 2021;20(3):318-35.
  - **○ ** This paper outlines how recent technological advances can enhance mental health care, the existing evidence for digital mental health tools, and key challenges and opportunities for real-world implementation.
- Weinstock LM, Bishop TM, Bauer MS, Benware J, Bossarte RM, Bradley J, et al. Design of a multicenter randomized controlled trial of a post-discharge suicide prevention intervention for high-risk psychiatric inpatients: The Veterans Coordinated Community Care Study. Int J Methods Psychiatr Res. 2024;33(4):e70003.
  - **○ ** A multicenter randomized controlled trial testing post-discharge CLASP outreach for reduction of suicide-related behaviors over six months compared with usual care in high-risk military veteran psychiatric inpatients as identified by validated machine learning algorithms.

## Data Availability

No datasets were generated or analysed during the current study.
